# Direct evidence of charge separation in a metal–organic framework: efficient and selective photocatalytic oxidative coupling of amines *via* charge and energy transfer†
†Electronic supplementary information (ESI) available: Materials and instrumentation, and supplemental figures and tables. See DOI: 10.1039/c7sc05296k


**DOI:** 10.1039/c7sc05296k

**Published:** 2018-02-20

**Authors:** Caiyun Xu, Hang Liu, Dandan Li, Ji-Hu Su, Hai-Long Jiang

**Affiliations:** a Hefei National Laboratory for Physical Sciences at the Microscale , CAS Key Laboratory of Soft Matter Chemistry , Collaborative Innovation Center of Suzhou Nano Science and Technology , Department of Chemistry , University of Science and Technology of China , Hefei , Anhui 230026 , P. R. China . Email: jianglab@ustc.edu.cn ; http://staff.ustc.edu.cn/∼jianglab/; b CAS Key Laboratory of Microscale Magnetic Resonance , Synergetic Innovation Center of Quantum Information and Quantum Physics , Department of Modern Physics , University of Science and Technology of China , Hefei 230026 , P. R. China

## Abstract

The selective aerobic oxidative coupling of amines under mild conditions is an important laboratory and commercial procedure yet a great challenge. In this work, a porphyrinic metal–organic framework, PCN-222, was employed to catalyze the reaction. Upon visible light irradiation, the semiconductor-like behavior of PCN-222 initiates charge separation, evidently generating oxygen-centered active sites in Zr-oxo clusters indicated by enhanced porphyrin π-cation radical signals. The photogenerated electrons and holes further activate oxygen and amines, respectively, to give the corresponding redox products, both of which have been detected for the first time. The porphyrin motifs generate singlet oxygen based on energy transfer to further promote the reaction. As a result, PCN-222 exhibits excellent photocatalytic activity, selectivity and recyclability, far superior to its organic counterpart, for the reaction under ambient conditions *via* combined energy and charge transfer.

## Abstract 2

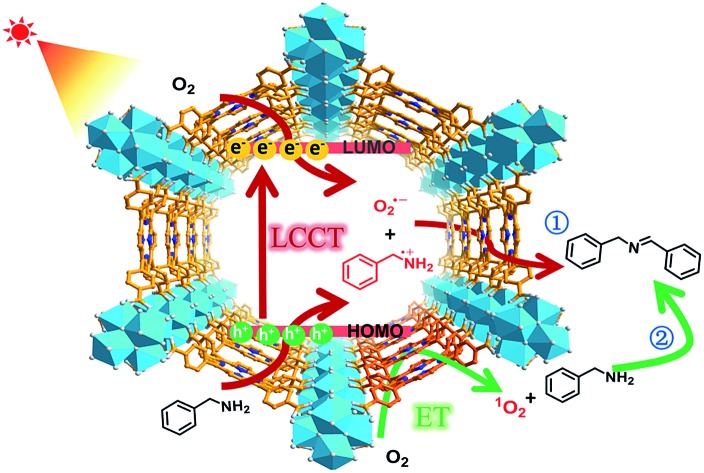
For the first time, the photoexcited charge separation in a metal–organic framework has been evidenced with clear ESR signals, based on efficient and selective photocatalytic oxidative coupling of amines.

## Introduction

Efficient and selective oxidation reactions based on molecular oxygen (O_2_) or even ambient air as the oxidant have received tremendous attention from the view of green chemistry. In particular, the selective oxidative coupling of amines to imine derivatives as important building blocks for the synthesis of fine chemicals and pharmaceuticals is highly desired.^1^ Diverse metal complex catalysts have been reported toward the aerobic oxidative coupling of amines. Unfortunately, relatively high temperature (∼373 K) and/or oxygen pressure (∼5 atm) are required to proceed.^1^,^2^ Alternatively, photo-induced aerobic oxidation emerges to be an effective and sustainable approach under moderate conditions. Photosensitizer molecules absorb sunlight to generate singlet oxygen (^1^O_2_) that can promote amine oxidative coupling. However, with ^1^O_2_ as a sole oxidant, the reaction efficiency needs to be improved and homogeneous systems are associated with intrinsic difficulty of catalyst separation and/or intermolecular deactivation.^3^ In contrast, heterogeneous semiconductor photocatalysts possess excellent catalytic performance for the aerobic oxidative coupling of amines, in which photogenerated electrons transferring to O_2_ to give superoxide radicals (O_2_˙^–^) and amines activated by the remaining holes are proposed. Though O_2_˙^–^ is detectable *via* the electron spin resonance (ESR) technique, actually, the oxidation product has never been evidenced and the reaction mechanism has remained disputable in heterogeneous catalytic systems thus far.^4^ Moreover, given the strong oxidation ability of holes, semiconductor photocatalysts usually produce aldehyde byproducts, lowering the selectivity to imines.^5^ Therefore, the exploration of a specific mechanism regarding this reaction over semiconductor-like photocatalysts for excellent activity and selectivity is imperative.

Metal–organic frameworks (MOFs) are a class of crystalline porous materials and exhibit potential applications in diverse fields.^6^–^8^ The well-defined structures constructed from metal (oxide) nodes and organic linkers endow MOFs with semiconductor-like behavior for photocatalysis.^7^,^8^ Upon light irradiation, different processes might occur in MOFs, such as ligand-to-metal charge transfer (LMCT), metal-to-ligand charge transfer (MLCT), π–π* transition of the delocalized linker, *etc.*^8^ However, only a half-reaction radical intermediate, usually electron-reduced metal ions, was detected in previous reports, while the radical cation (*e.g.*, excited ligand) cannot be recognized, possibly due to its very short lifetime. Solid evidence collection for both electron- and hole-involved intermediates in MOF photocatalysts remains a highly desired goal to understand charge separation in MOFs.

**Scheme 1:**
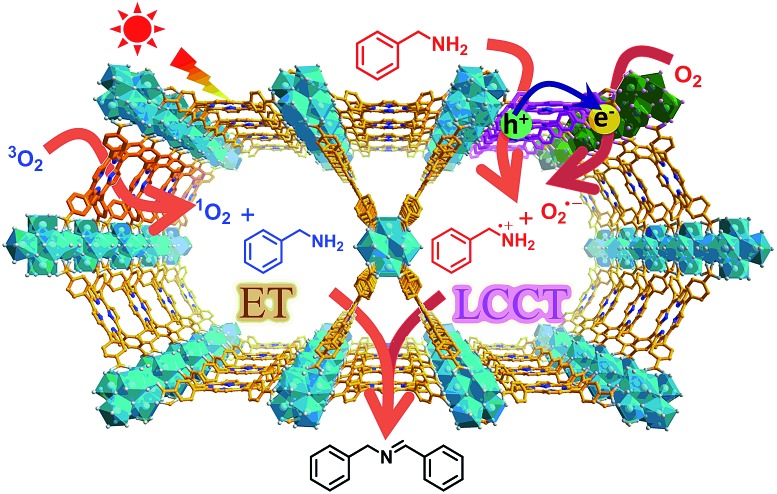
Schematic illustration of LCCT and ET processes involved in PCN-222 for the selective oxidative coupling of amines under visible light irradiation.

In this work, a representative porphyrinic MOF, PCN-222 (also known as MOF-545 or MMPF-6),^9^ was chosen for the aerobic oxidative coupling of amines under visible light and ambient conditions. The porphyrin linkers in PCN-222 can be excited by visible light and the photogenerated electrons transfer to Zr-oxo clusters to form oxygen-centered active sites, leaving enhanced porphyrin π-cation radical signals by hole oxidation, both of which have been detected *via* ESR. The results clearly manifest the linker-to-cluster charge transfer (LCCT) procedure, accounting for the semiconductor-like behavior of PCN-222. The electrons and holes further transfer and produce O_2_˙^–^ and benzylamine radicals (PhCH˙NH_2_ and PhCH_2_NH˙), respectively, which have been directly evidenced in heterogeneous photocatalytic systems for the first time, synergistically promoting the selective formation of target imines. In addition, the energy transfer (ET) gives rise to ^1^O_2_ generation over porphyrin motifs by light irradiation. The combination of charge and energy transfer processes in PCN-222 leads to its excellent catalytic activity and selectivity under ambient conditions (Scheme 1).

## Results and discussion

The PCN-222, Zr_6_(μ_3_-OH)_8_(OH)_8_(TCPP)_2_ (TCPP = tetrakis(4-carboxyphenyl)porphyrin), was selected as it features a 3D structure with large channels larger than 3 nm and a high BET surface area of 1659 m^2^ g^–1^ (Fig. S1†). Importantly, it exhibits a strong UV-Vis response in the 300–800 nm range, inheriting the Soret band (∼420 nm) and four Q bands (500–700 nm) from the porphyrin ligand (Fig. S2†). The Soret band and Q bands are deemed to originate from two different transitions from the HOMOs (a_1u_ and a_2u_) to the LUMO (e_g_),^10^ indicating the similar electronic structure of PCN-222 and the porphyrin molecule TPPCOOMe. This hypothesis is supported by density functional theory (DFT) calculations (Fig. S3 and S4†) and cyclic voltammetry measurements (Fig. S5†). Therefore, behaving like a semiconductor, PCN-222 can be excited by visible light and the photogenerated electrons can transfer from the porphyrin ligand to Zr-oxo clusters, where the HOMO and LUMO are located, respectively.7*g* Based on the UV-Vis spectra and electrochemical characterization of PCN-222 and TPPCOOMe, their energy diagrams of the HOMO–*n*, HOMO and LUMO can be clearly given (Fig. S6†).^11^ Given the more negative potential of the LUMO level (–0.39 V *vs.* NHE) in PCN-222 than the reduction potential of O_2_ to related reactive oxygen species (ROS, *E*(O_2_/O_2_˙^–^) = –0.33 V *vs.* NHE),10*a* it is theoretically feasible for photocatalytic O_2_˙^–^ generation. The porphyrin ligand would act as a visible-light-harvesting unit and contribute to the HOMO and HOMO–*n* at +1.43 V and +2.20 V *vs.* NHE, respectively, calculated by using the Tauc plot (Fig. S6†).^11^ The relatively lower occupied molecular orbital level guarantees a high oxidation ability for benzylamine (*E*_1/2_(M^+^/M) of ∼+1.47 V *vs.* NHE).^12^

The above results encourage us to investigate the oxidative coupling of benzylamines over PCN-222 in air under visible light irradiation (Table 1). Acetonitrile was found to be the most suitable medium among different solvents examined (Table S1†). Benzylamine can be completely converted to the corresponding target product, *N*-benzylidenebenzylamine, in 1 h over PCN-222, where light irradiation and air atmosphere are necessary (entries 1–4). A higher activity can be achieved under an O_2_ atmosphere (entry 5). Moreover, this catalyst exhibits excellent activity under simulated solar irradiation (entry 6), extending its potential to practical application. Upon gradually increasing the light intensity, the conversion increases accordingly, reflecting the photo-driven process (Fig. S7†). In contrast, the active motif, 5,10,15,20-tetrakis(4-methoxycarbonyl phenyl)porphyrin (TPPCOOMe), gives only a 26.7% yield and a much lower activity than PCN-222 with a fixed porphyrin amount (entry 7; Fig. S8†), which indicates that the porphyrin ligand itself cannot give the high efficiency of PCN-222. The stability and reusability of PCN-222 were examined and its activity was maintained very well in five consecutive runs (Fig. S9†). After that, the powder X-ray diffraction pattern of PCN-222 revealed its structural integrity and stability (Fig. S10†). After 30 min of reaction, the catalyst was filtered out and the results showed that no further product was formed even after 1.5 h (Fig. S11†), indicating that the process should be truly heterogeneous. Therefore, PCN-222 possesses truly heterogeneous nature with excellent activity, selectivity, stability and recyclability toward the photocatalytic oxidative coupling of benzylamine.

**Table 1:** Oxidative coupling of benzylamines under various conditions^*a*^

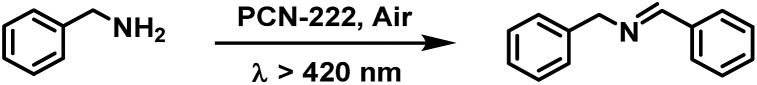					
Entry	Catalyst	Air	Light	*t* [h]	Conv.^*b*^ [%]
1	PCN-222	+	+	1	100
2	—	+	+	2	1
3	PCN-222	+	–	2	—
4^*c*^	PCN-222	–	+	1	44.4
5^*d*^	PCN-222	–	+	0.75	100
6^*e*^	PCN-222	+	+	1.5	95.6
7^*f*^	TPPCOOMe	+	+	1	26.7

^*a*^Reaction conditions: 0.1 mmol benzylamine, 5 mg PCN-222, 100 mW cm^–2^ Xe lamp cutoff below 420 nm, 3 mL CH_3_CN, and air atmosphere.

^*b*^Determined by GC analysis.

^*c*^Under a 1 atm N_2_ atmosphere.

^*d*^Under a 1 atm O_2_ atmosphere.

^*e*^Under an AM 1.5 illumination of 100 mW cm^–2^.

^*f*^3.6 mg TPPCOOMe.

Upon the optimization of reaction conditions by using benzylamine as a probe substrate, to demonstrate the general applicability of PCN-222, different amines have been examined (Table 2). Primary benzylamine derivatives substituted with various functional groups were converted to corresponding imines with excellent conversions, in which the oxidation of the benzylamines with electron-donating groups proceeded slightly faster than those with electron-withdrawing groups (entries 1–8). The reaction rate of the regioisomers decreased in the *para* > *meta* > *ortho* order, indicating the presence of the steric effect (entries 2–4).4*a* Heterocyclic amines containing N, O and S atoms, which usually poison metal catalysts,4*a* can be well tolerated and displayed excellent yields (entries 9–11). Moreover, both secondary and cyclic amines were amenable to the procedure, giving the corresponding imines in satisfactory yields (entries 12 and 13). However, the oxidative coupling of aniline without a hydrogen atom at its α-carbon did not proceed (entry 14), suggesting that reaction might occur *via* hydrogen abstraction.

**Table 2:** Photocatalytic oxidative coupling of various amines into imines^*a*^

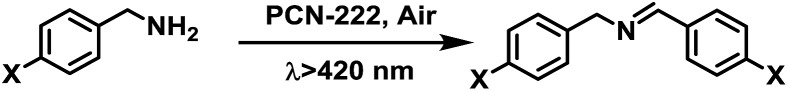					
Entry	Substrate	Product	*t* [min]	Conv.^*b*^ [%]	Sel.^*b*^ [%]
1	X = H	X = H	60	100	100
2	X = *o*-OCH_3_	X = *o*-OCH_3_	65	100	100
3	X = *m*-OCH_3_	X = *m*-OCH_3_	55	100	100
4	X = *p*-OCH_3_	X = *p*-OCH_3_	45	100	100
5	X = *p*-CH_3_	X = *p*-CH_3_	60	100	100
6	X = *p*-F	X = *p*-F	70	100	100
7	X = *p*-Cl	X = *p*-Cl	65	100	100
8	X = *p*-CF_3_	X = *p*-CF_3_	75	100	100
9	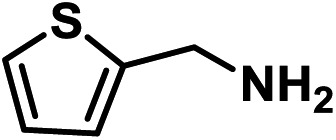	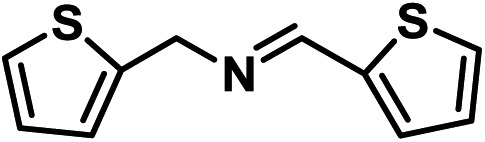	80	100	100
10	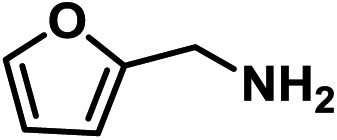	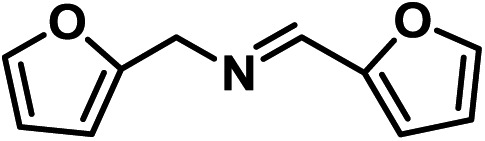	65	100	100
11	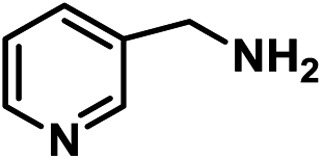	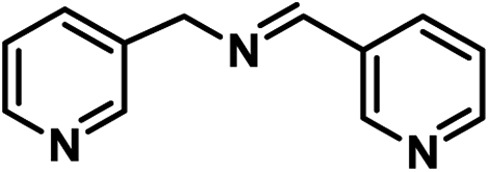	60	100	100
12	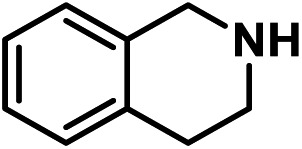	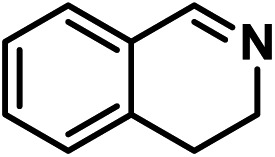	70	100	100
13	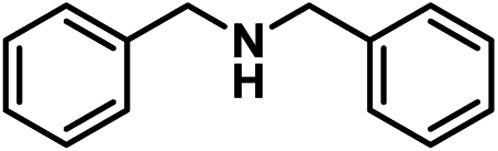	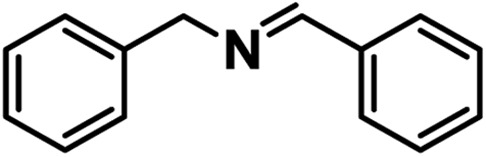	60	100	100
14	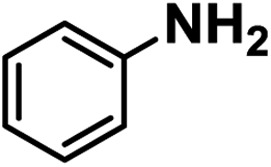	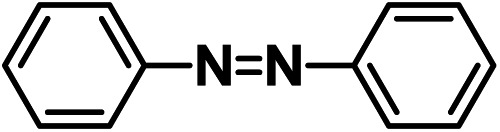	120	—	—

^*a*^Reaction conditions: 0.1 mmol amine, 5 mg PCN-222, 100 mW cm^–2^ Xe lamp cutoff below 420 nm, 3 mL CH_3_CN, and air atmosphere.

^*b*^Determined by GC-MS.

**Fig. 1:**
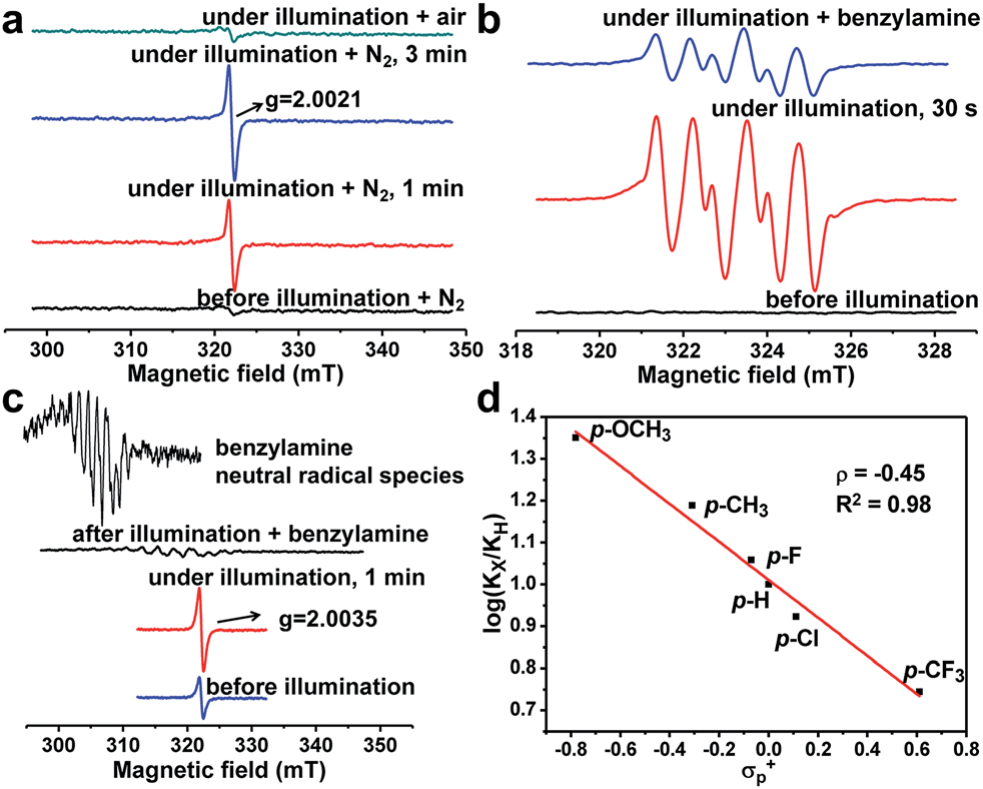
(a) ESR detection of the electron transfer process in PCN-222. (b) ESR detection of O_2_˙^–^ generation over PCN-222 trapped by DMPO. (c) The detection of hole-involved intermediates in PCN-222. (d) Hammett plot for the oxidation of benzylamine and its *para*-substituted derivatives photocatalyzed by PCN-222. All ESR tests were described in detail in the Experimental section.

It is necessary to elucidate the reaction mechanism behind the above excellent photocatalytic performance of PCN-222. The light-induced porphyrin π-cation radical was monitored *in situ* by using the ESR technique (Fig. S12–S14†). Interestingly, the porphyrin ligand (TPPCOOMe) shows an unchanged ESR signal while PCN-222 gives a much enhanced porphyrin π-cation radical signal upon light irradiation, as shown by the ESR light–dark difference spectra (Fig. S13 and S14†), indicating the generation of photogenerated radicals (*i.e.*, e–h pairs) in PCN-222, compared to porphyrin molecules.10*a* Hence, the effective electron transfer in PCN-222 results in the remaining holes contributing to the enhanced ESR signal of cation radicals. The electron transfer destination from the ligand in PCN-222 can be deduced by using the ESR technique as well. A new ESR signal at *g*_iso_ = 2.0021 (close to the value of free electrons, *g*_e_-2.0023) emerged and its signal intensity strengthened with a prolonged irradiation time (Fig. 1a), which might originate from oxygen-centered active sites in Zr-oxo clusters.^13^ The above results demonstrate that photo-generated electrons transfer from the porphyrin ligand to Zr-oxo clusters, generating oxygen-centered active sites in Zr-oxo clusters and porphyrin π-cation radicals in the ligand, which, unprecedentedly provide direct evidence for the LCCT procedure in a MOF.

More evidence has been gleaned to track the transfer pathway of photo-generated carriers occurred in PCN-222 and reactants. Upon exposure to O_2_/air, the signal at *g* = 2.0021 was eliminated (Fig. 1a), indicating that the formed active sites possess reducibility and are able to reduce O_2_ to O_2_˙^–^ species (shown below further). It has been well established that *t*-butanol and 1,4-benzoquinone (BQ, an known electron inhibitor) can effectively inhibit the generation of OH˙ and O_2_˙^–^, respectively,^14^ in which only BQ can effectively inhibit the reaction (Fig. S15†), possibly due to the high oxidation potential of *E*(–OH/˙OH) (+2.80 V *vs.* NHE) that is insufficient for the HOMO–*n* of PCN-222.^10^ No target product was detected by introducing H_2_O_2_ (Table S2†), indicating that it is not the ROS. In addition, 5,5-dimethyl-pyrroline-*N*-oxide (DMPO) was employed as the O_2_˙^–^-sensitive trapping agent, further manifesting the O_2_˙^–^ formation over PCN-222 (Fig. 1b).4*a* Upon the addition of benzylamine, the signal intensity of DMPO-O_2_˙^–^ evidently decreases, suggesting the strong interaction between benzylamine and O_2_˙^–^. These results jointly point out that electron transfer occurs between Zr-oxo clusters and O_2_ to generate O_2_˙^–^ as the ROS for the subsequent oxidative coupling of amines. In addition, the photocatalytic oxidation rates of benzylamine and its *para*-substituted derivatives (X = OMe, Me, H, F, Cl, and CF_3_ groups) were examined over PCN-222 to obtain the Hammett plot (Fig. 1d). A reasonable linearity between log(*k*_x_/*k*_h_) values and Brown–Okamoto constant (*σ*_p_^+^) parameters^15^ was obtained with a negative slope, indicating the hole transfer from porphyrin π-cation radicals in PCN-222 to benzylamine to give a positively charged intermediate, possibly PhCH_2_NH_2_˙^+^.4*a* Once benzylamine was injected, the porphyrin π-cation radical ESR signal was quenched immediately; a set of weak signals simultaneously emerged that can be assigned to the combined signals of benzyl-type carbon-centered (PhCH˙NH_2_) and nitrogen-centered (PhCH_2_NH˙) radicals (Fig. 1c, S16 and S17†), generated from the hydrogen abstraction from PhCH_2_NH_2_˙^+^,^16^ indicative of hole transfer from porphyrin π-cation radicals to benzylamine.

The specific catalytic sites in the charge transfer process were further determined by the analysis of the energy diagram of PCN-222. The steady-state photoluminescence (PL) spectra show that the excitation of the Soret band (*λ* = 420 nm) leads to much lower PL intensity in PCN-222, which indicates the rapid and effective charge transfer occurred in this process (Fig. S2 and S18†).8*e* In contrast, the PL is stronger than that of TPPCOOMe upon the excitation of Q bands (*λ* = 520 nm). The results clearly support the previous argument that the transition from the HOMO–*n* to the LUMO in PCN-222 mainly contributes to the benzylamine oxidation. Further photocurrent tests also prove this point, as the photocurrent under a single excitation wavelength (*i.e.*, *λ* = 420 nm) accounts for a greater portion of the total photocurrent (Fig. S19b†).

**Fig. 2:**
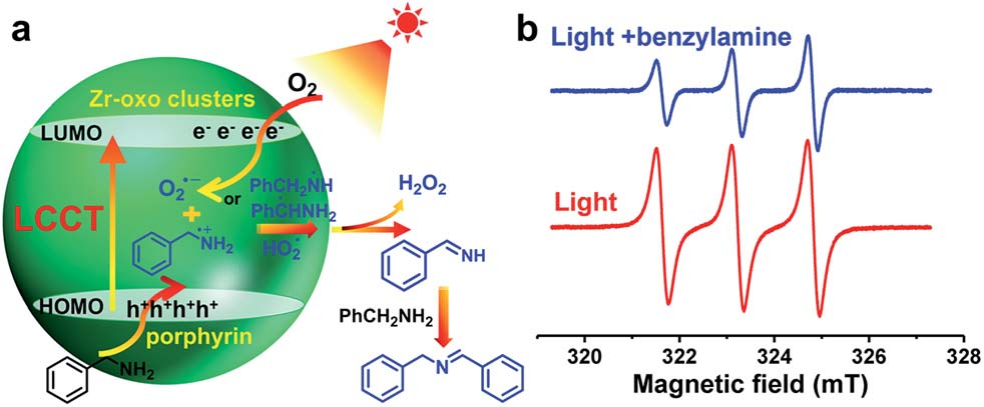
(a) Proposed charge transfer process for the photocatalytic oxidative coupling of benzylamine over PCN-222, in which the complex HOMO constitution is simplified. (b) ESR spectra (light–dark difference) of PCN-222 for the ^1^O_2_ detection in the presence of 4-oxo-TMP.

With the above data in hand, the charge transfer mechanism in PCN-222 for the photocatalytic oxidative coupling of benzylamine has been proposed (Fig. 2a). Upon irradiation, the LCCT process mainly from the HOMO–*n* to the LUMO in PCN-222 gives rise to effective e–h separation, which reduces O_2_ to O_2_˙^–^ and oxidizes benzylamine to PhCH_2_NH_2_˙^+^, respectively. Thanks to the synergistic effect of electron–hole pairs, proton transfer from PhCH_2_NH_2_˙^+^ to O_2_˙^–^ takes place under mild conditions, generating hydrogen abstracted benzylamine radicals (PhCH˙NH_2_ and PhCH_2_NH˙) and hydroperoxyl radicals (HO_2_˙), which are converted to benzylideneamine (PhCH=NH) and H_2_O_2_. The H_2_O_2_ byproduct has been detected in the reaction system *via* a well-established method (Fig. S20†). The PhCH=NH further reacts with benzylamine to produce the target product.^16^

In addition to the O_2_˙^–^ species generated during the charge transfer process, photosensitized porphyrin motifs can react with ground state oxygen (^3^O_2_) to generate ^1^O_2_*via* triplet–triplet annihilation (TTA) in PCN-222.14*b* To evidence ^1^O_2_ generation, 4-oxo-TMP, the classical ^1^O_2_ probe, was employed to display intense characteristic signals (*g* = 2.0055, 1 : 1 : 1 triplet) of 4-oxo-TEMPO under light irradiation for PCN-222 (Fig. 2b and S21†). The signals evidently decreased upon the addition of benzylamine, manifesting that ^1^O_2_ indeed participates in the reaction. The addition of an ^1^O_2_ scavenger remarkably impaired the conversion (Fig. S15†), again supporting the ^1^O_2_ production. The generated ^1^O_2_ based on the energy transfer process featuring high electrophilicity would directly oxidize amines to imines.^3^,^12^ Therefore, the combination of O_2_˙^–^ generation *via* charge transfer and ^1^O_2_ production *via* energy transfer contributes to the ultimate great activity of PCN-222 (Scheme 1 and Fig. S22†).

To identify the ROS that are responsible for the activity of TPPCOOMe, scavenging experiments were conducted on the ligand. Among different ROS scavengers, the conversion can be completely inhibited in the presence of an ^1^O_2_ scavenger (Fig. S23†). In sharp contrast to that of PCN-222, the reaction over TPPCOOMe was almost insusceptible to the introduction of ˙OH and O_2_˙^–^ scavengers and less affected by hole scavengers (Fig. S24†). The characteristic ^1^O_2_ ESR signal intensity for TPPCOOMe is similar to that for PCN-222 (Fig. S25†), while the signal intensity of O_2_˙^–^ radicals in TPPCOOMe is much weaker (Fig. S26†), due to the much lower charge separation efficiency in the organic entity (Fig. S18a and S19a†). Given the above results, compared with the cooperative effect of O_2_˙^–^ and ^1^O_2_ in the reaction over PCN-222, ^1^O_2_ being the nearly sole ROS accounts for the poor activity of TPPCOOMe (Table 1, entry 7). In addition, there is no obvious linear correlation between log(*k*_x_/*k*_h_) and *σ*_p_^+^ in the Hammett plot (Fig. S27†), indicating the lack of positively charged intermediates produced by hole activation for the process over TPPCOOMe.

## Experimental section

### Synthesis of the porphyrin ligand (H_2_TCPP)

The tetrakis(4-carboxyphenyl)porphyrin (H_2_TCPP) ligand was synthesized according to the previous report with minor modifications.^17^ The typical synthetic procedures are described below.

#### Synthesis of 5,10,15,20-tetrakis(4-methoxycarbonylphenyl)-porphyrin (TPPCOOMe)

Pyrrole (3.0 g, 44.7 mmol) and methyl 4-formylbenzoate (7 g, 42.6 mmol) were added to 100 mL of propionic acid in a 250 mL three necked flask and the solution was refluxed for 12 h at 140 °C. The reaction flask was then transferred to a refrigerator for five hours. The reaction solution was subjected to filtration, and then washed with 200 mL of ethanol, 100 mL of ethyl acetate and 50 mL of THF to remove black impurities, and finally dried in a vacuum.

#### Acidification of TPPCOOMe to form H_2_TCPP

The obtained ester TPPCOOMe (1.95 g) was dissolved in a mixed solvent of THF (60 mL) and MeOH (60 mL). A solution of KOH (6.82 g, 122 mmol) in H_2_O (60 mL) was introduced slowly into the above system. This mixture was then refluxed at 85 °C for 12 h. The obtained homogeneous solution was diluted with water and acidified with 1 M HCl until a purple solid was precipitated. The purple solid was collected by filtration, washed with water and dried in a vacuum.

### Synthesis of PCN-222

The MOF was synthesized based on the previous report with some modifications.^17^ Typically, ZrOCl_2_·8H_2_O (108.6 mg), H_2_TCPP (30 mg) and CF_3_COOH (0.45 mL) were ultrasonically dissolved in 10 mL of DMF in a 20 mL Pyrex vial. The mixture was heated at 120 °C in an oven for at least 16 h. Purple needle shaped crystals were harvested by filtration and washed with DMF and acetone, respectively. Prior to use, the solid was soaked in 100 mL acetone for 48 h to exchange out DMF, and then filtered and dried in a vacuum.

### Activity evaluation on photocatalytic oxidative coupling of amines

In a typical procedure, 5 mg of PCN-222 or 3.6 mg of TPPCOOMe as a photocatalyst was dispersed in 3 mL of solvent, followed by the addition of 0.1 mmol of amine substrate in a 5 mL Pyrex vial equipped with a rubber septum and a tiny syringe needle connected the reaction system with the surrounding air atmosphere outside. The sample was stirred vigorously and irradiated with a 300 W xenon lamp equipped with an ultraviolet-cutoff filter (cutting out light below 420 nm) at a fixed light intensity of 100 mW cm^–2^ unless otherwise stated. Then, the conversion was quantified by gas chromatography and the products were confirmed by GC-MS after centrifugation. The mass of TPPCOOMe was calculated based on the equivalent amount of porphyrin motifs in PCN-222. The optimized reaction conditions for the photocatalytic oxidative coupling reaction of amines were determined, which consist of 5 mg PCN-222, 0.1 mmol amine, 3 mL CH_3_CN, under a Xe lamp cutoff below 420 nm at a light intensity of 100 mW cm^–2^ and ambient conditions (in the surrounding air atmosphere).

#### Recyclability of oxidative coupling of benzylamine over PCN-222

After the reaction indicated above, the reaction solution was centrifuged at 12 000 rpm for 2 min after each cycle and washed with CH_3_CN 2 times. Then the catalyst was reused for the subsequent run with fresh benzylamine (0.1 mmol) under the optimized reaction conditions.

### ESR measurements

ESR was recorded on a JES-FA200 electron paramagnetic resonance spectrometer under visible-light irradiation (*λ* > 400 nm). The *in situ* ESR tests were conducted with a homemade tube with a special setup (Fig. S12†).

#### ESR detection of porphyrin π-cation radicals in PCN-222 and TPPCOOMe

The variation in temperature, atmosphere, and illumination was previously demonstrated to have no influence on the porphyrin π-cation radical ESR signal for porphyrin molecules.^18^ ESR measurements were carried out with 5 mg of PCN-222 or 3.6 mg of TPPCOOMe solid powder under irradiation with a 500 W xenon lamp (*λ* > 400 nm) at 140 K under open air conditions in a quartz cube.

#### ESR analysis of the electron transfer process in PCN-222

For a better detection of the electron transfer pathway in PCN-222, the hole sacrificial agent TEOA was added into the system before testing. Typically, 10 μL of TEOA was injected into 300 μL of PCN-222/acetonitrile suspension (5 mg/3 mL). ESR measurements were carried out under irradiation with a 500 W xenon lamp (*λ* > 400 nm) at 140 K under a N_2_ atmosphere in a sealed homemade ESR tube with an oxygen balloon. During the irradiation process, the three-way valve was opened to introduce oxygen into the tube as required.

#### ESR detection of benzylamine intermediates

For a better detection of the hole transfer pathway in PCN-222, an electron scavenger AgNO_3_ was added into the system before testing. Typically, 20 mg of AgNO_3_ was dissolved in a PCN-222/acetonitrile suspension (5 mg/3 mL) and then 300–400 μL of the mixture was injected into a quartz tube. ESR measurements were carried out under irradiation with a 500 W xenon lamp (*λ* > 400 nm) at 140 K in a sealed homemade tube. After illumination, the homemade ESR tube was taken out to thaw and then 20 μL of benzylamine was injected into the tube for subsequent examination at 140 K without light irradiation.

#### ESR detection of O_2_˙^–^ and ^1^O_2_ over PCN-222 and TPPCOOMe

1 mL of DMPO/toluene solution (30 μL/3 mL, for the detection of O_2_˙^–^) or 4-oxo-TMP/toluene solution (30 μL/3 mL, for ^1^O_2_) was mixed with 0.5 mL of PCN-222 (5 mg/3 mL) or TPPCOOMe/toluene suspension (3.6 mg/3 mL). The use of toluene solvent instead of acetonitrile is to avoid the disturbance of methyl radicals from acetonitrile. 300–400 μL of the mixture was added into an ESR tube. ESR measurements were carried out under irradiation with a 500 W xenon lamp (*λ* > 400 nm) at room temperature under open air conditions. For control experiments, 10 μL of benzylamine was added into the above testing system.

### Detection of hydrogen peroxide (H_2_O_2_)

The production of H_2_O_2_ was detected by a well-established *N*,*N*-diethyl-*p*-phenylenediamine (DPD)/horseradish peroxidase (POD) method.^19^ Typically, 100 mg of DPD was dissolved in 10 mL of H_2_SO_4_ (0.05 M) solution and 100 mg of POD was dissolved in 10 mL of distilled water, both of which were stored in the dark at below 278 K before use. The filtrate obtained after the photocatalytic oxidative coupling reaction of benzylamine over PCN-222 was added to 20 mL of H_2_O, and the mixture was further extracted by using ethyl acetate (EtOAc, 20 mL) three times to remove the organic compounds. Then the mixture was diluted to 400 mL with distilled H_2_O. 9 mL of the above aqueous solution was mixed with 1 mL of PBS buffer (pH = 7.4) and used as a test sample. After adding 20 μL of DPD and 20 μL of POD solution, the UV-Vis spectra of the sample were collected.

### Electrochemical characterization

#### Mott–Schottky plots

Mott–Schottky plots of PCN-222 were measured on an electrochemical workstation (Zahner Zennium) in a standard three-electrode system with a sample-coated electrode as the working electrode, a Pt plate as the counter electrode, and Ag/AgCl as the reference electrode at frequencies of 500, 1000, and 1500 Hz, respectively. A 0.1 M Na_2_SO_4_ deoxygenated solution was used as the electrolyte.

#### Cyclic voltammetry measurements

Cyclic voltammetry measurements were performed on a CHI 760E electrochemical workstation (Chenhua Instrument, Shanghai, China) in a standard three-electrode system with sample-coated glassy carbon (*Φ* = 3 cm) as the working electrode, a Pt plate as the counter electrode, and Ag/Ag^+^ as the reference electrode. Sodium alginate was selected as the binding agent. Both the samples have the same porphyrin content. A 0.1 M TBAF in CH_3_CN solution after deoxidation was used as the electrolyte. Ferrocene was used as an internal standard and the ferrocenium–ferrocene (Fc^+^/Fc) potential was used to correct potentials.

#### Photoelectrochemical measurements

Photoelectrochemical measurements were performed on a CHI 760E electrochemical workstation (Chenhua Instrument, Shanghai, China) in a standard three-electrode system with sample-coated ITO as the working electrode, a Pt plate as the counter electrode, and Ag/AgCl as the reference electrode. Nafion was used as the binding agent. Both the samples have the same porphyrin content. A xenon lamp with different optical filters was used as the light source. The photoresponse signals of the samples were measured under chopped light of different wavelengths without an extra bias potential.

### Photoluminescence (PL) spectra

Typically, 2 mg PCN-222 (or 1.44 mg TPPCOOMe, a fixed content of porphyrin) was dispersed in 6 mL CH_3_CN. Steady-state PL spectra were recorded by using a PerkinElmer LS 55 Fluorescence Spectrometer.

### DFT calculations

Density functional theory (DFT) calculations were carried out *in vacuo* for a better understanding of charge transfer. Optimizations were carried out with B3LYP[LANL2DZ] without any symmetry restraint. All calculations were performed using G03 software. The geometry optimization of the singlet ground state was performed with a basis set composed of 6-31 G* for C, H, N, and O atoms.

## Conclusions

In summary, the visible-light photocatalytic oxidative coupling of diverse amines has been promoted by using a porphyrinic MOF (PCN-222) under ambient conditions. In addition to the direct oxidative coupling of amines *via*^1^O_2_ that is generated by the porphyrin ligand based on energy transfer, the synergistic effect between charge transfer-induced electrons and holes greatly contributes to the reaction. As a result, PCN-222 exhibits excellent photocatalytic activity, selectivity and recyclability toward this reaction, far superior to its corresponding TPPCOOMe counterpart that involves an energy transfer process only. The photoexcited PCN-222 produces oxygen-centered active sites in Zr-oxo clusters and porphyrin π-cation radicals in the reduction and oxidation ends, respectively, verified by ESR, which, for the first time, afford direct evidence for the charge separation and semiconductor-like behavior of MOFs. Moreover, the electron- and hole-engaged reaction intermediates have been clearly identified by ESR, which has remained unprecedented thus far, to understand the mechanism based on charge transfer for this reaction. This work not only provides an insightful understanding for charge transfer/separation in MOFs but would also stimulate further studies on photocatalytic organic transformation over MOFs.

## Conflicts of interest

There are no conflicts to declare.

## Supplementary Material

Supplementary information
