# Supplementary material for: Direct evidence of charge separation in a metal–organic framework: efficient and selective photocatalytic oxidative coupling of amines via charge and energy transfer
Source: Chem Sci. 2018 Feb 20;9(12):3152–8. doi: 10.1039/c7sc05296k (PMC5916110; doi:10.1039/c7sc05296k)
Supplement: Supplementary file 1 [file SC-009-C7SC05296K-s001.pdf]

Electronic Supplementary Material (ESI) for Chemical Science.
This journal is © The Royal Society of Chemistry 2018

Electronic Supplementary Information

Direct Evidences on Charge Separation in a Metal-Organic Framework: Efficient and Selective Photocatalytic Oxidative Coupling of Amines via Charge and Energy Transfer

Caiyun Xu,[‡a] Hang Liu,[‡a] Dandan Li,[a] Ji-Hu Su,[b] and Hai-Long Jiang[*,a]

[a] Hefei National Laboratory for Physical Sciences at the Microscale, CAS Key Laboratory of Soft Matter Chemistry, Collaborative Innovation Center of Suzhou Nano Science and Technology, Department of Chemistry, [b] CAS Key Laboratory of Microscale Magnetic Resonance, Synergetic Innovation Center of Quantum Information and Quantum Physics, Department of Modern Physics, University of Science and Technology of China, Hefei, Anhui 230026, P.R. China

‡These authors contributed equally to this work.

*To whom correspondence should be addressed.

E-mail: jianglab@ustc.edu.cn (H.L.J.)

S1 Materials and Instrumentation

All chemicals were obtained from commercial sources and used without further purification. Zirconyl chloride octahydrate ($ZrOCl_2 \cdot 8H_2O$, 98%), pyrrole (CP), and triethylamine (TEA, AR) were purchased from Aladdin Industrial Inc. N,N-dimethylformamide (DMF, AR), propionic acid (CH_3CH_2COOH, AR), tetrahydrofuran (THF, AR), KOH (AR), hydrochloric acid (HCl, AR), aniline (AR), *t*-butanol (CP), and triethanolamine (TEOA, AR) were purchased from Sinopharm Chemical Reagent Co., Ltd. Trifluoroacetic acid (CF_3COOH, 99%), methyl 4-formylbenzoate (99%), 1,4-benzoquinone (BQ, 98%) benzylamine (BA, 98%) dibenzylamine (99%), 4-fluorobenzylamine (98%), 4-methoxybenzylamine (98%), 2-picolylamine (98%), 2-thiophenemethylamine (97%), $AgNO_3$ (99.8%), hydrogen peroxide (H_2O_2, 30%), tetrabutylammonium fluoride (TBAF, 98%), and 2-methylfuran (98%) were purchased from Energy Chemical. Ethanol (EtOH, AR), ethyl acetate (AR), and methanol (MeOH, AR) were purchased from General Reagent. The 4-methylbenzylamine (98%) was purchased from Macklin Inc. The furfuryl amine (99%), 1,2,3,4-tetrahydroisoquinoline (97%), 3-methoxybenzylamine (98.5%), 4-(trifluoromethyl)benzylamine (98%), 4-chlorobenzylamine (98%), and 5,5-dimethyl-1-pyrroline N-oxide (DMPO, 97%) were purchased from J&K. The 2-methylbenzylamine (97%) and 2,2,6,6-tetramethyl-4-piperidone hydrochloride (4-oxo-TMP, 98%) were from TCI.

De-ionized water was prepared by reversed osmosis (the specific resistance of 18.25 MΩ·cm) followed by ion-exchange and filtration (Cleaned Water Treatment

Co., Ltd., Hefei). Powder X-ray diffraction patterns (PXRD) were acquired on a Japan Rigaku SmartLabTM rotation anode X-ray diffractometer or Holland X'Pert PRO fixed anode X-ray diffractometer equipped with graphite monochromatized Cu Kα radiation (λ = 1.54 Å). Gas sorption measurement was conducted by using an automatic volumetric adsorption equipment (Micrometritics ASAP 2020). UV-Vis absorption spectrum was recorded on a Shimadzu UV-2700 spectrophotometer and a white standard of $BaSO_4$ was used as a reference. The catalytic reaction products were identified and analyzed by using a Shimadzu gas chromatograph (GC-2010 Plus with a 0.25 mm × 30 m Rtx®-5 capillary column). The products were determined by using the Agilent technologies 7890A GC and a mass spectrometer Agilent technologies 5975C as detector. The light irradiation was obtained by 300 W Xenon lamp (LX300F, Japan) otherwise stated.

S2 Experimental Section

Table S1. The solvent influence on the oxidative coupling of benzylamine over PCN-222.

Entry	Solvent	Conversion (%)	Selectivity (%)
1	CH_3OH	20.1	100
2	CH_3CH_2OH	26.1	100
3	DMF	56.2	100
4	CH_3CN	82.8	100
5	toluene	71.8	100
6	1,4-dioxane	89.3	73

Reaction conditions: 0.1 mmol benzylamine, 5 mg PCN-222, 100 mW/cm^2 Xe lamp cutoff below 420 nm, 3 mL solvent, reaction time of 45 min and open-air conditions.

Table S2. The H_2O_2 influence on the conversion of benzylamine.[a]

Entry	Catalyst	H_2O_2 (mM)	Air	T (°C)	Light	t (min)	Conv. (%)[b]
1	-	5	+	50	-	60	1.2
2	PCN-222	5	+	r.t.	-	60	1.1
3	PCN-222	5	+	50	-	60	1.2
4[c]	PCN-222	5	-	r.t.	+	38	10.1
5[c]	PCN-222	5	-	r.t.	+	60	17.9
6[c]	PCN-222	-	-	r.t.	+	60	44.4

[a] Reaction conditions: 0.1 mmol of benzylamine, 5 mg of PCN-222, 3 mL of CH_3CN, 5 mM H_2O_2 and air atmosphere. [b] Determined by GC analysis. [c] Under N_2 atmosphere, 100 mW/cm^2 Xe lamp cutoff below 420 nm.

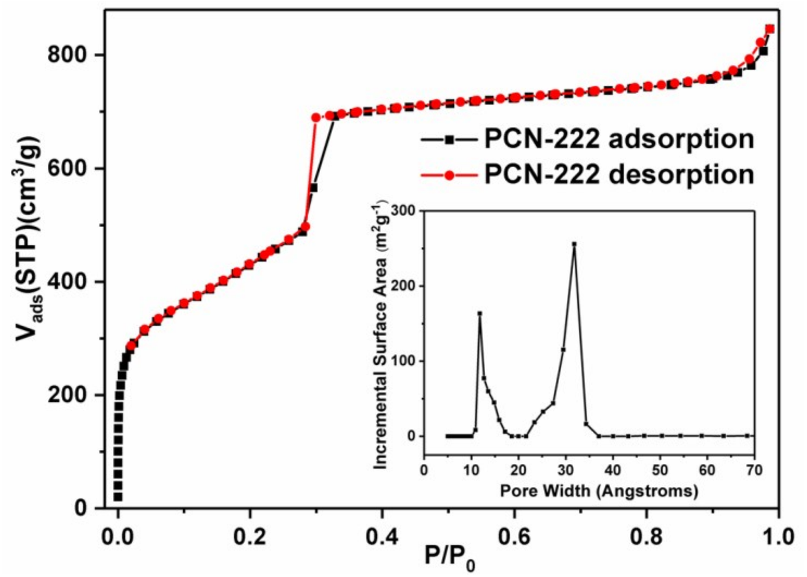

Fig. S1 N_2 sorption isotherms for PCN-222 at 77 K. Inset: pore size distribution for PCN-222 based on DFT.

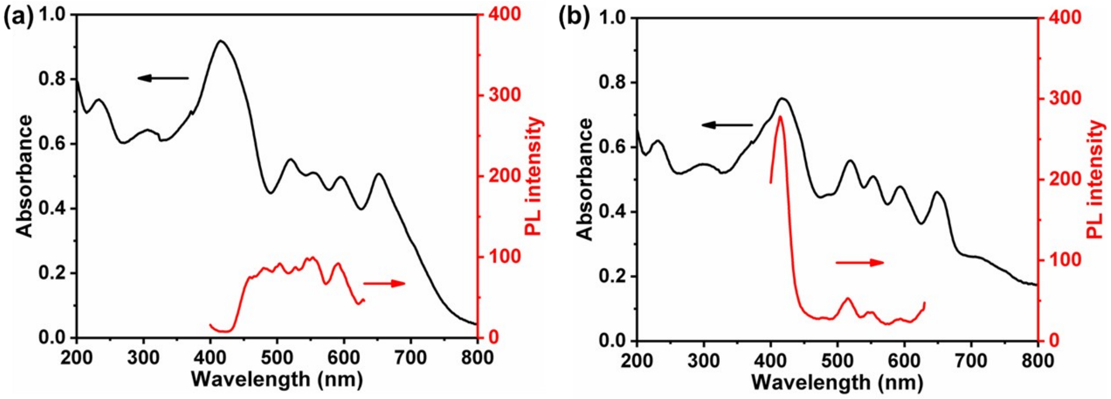

Fig. S2 UV-Vis absorption spectra (black line) and photoluminescence excitation spectra (red line) for (a) PCN-222 and (b) TPPCOOMe under λ_{em} = 650 nm, which is decided by the emission spectra (see Fig. S18).

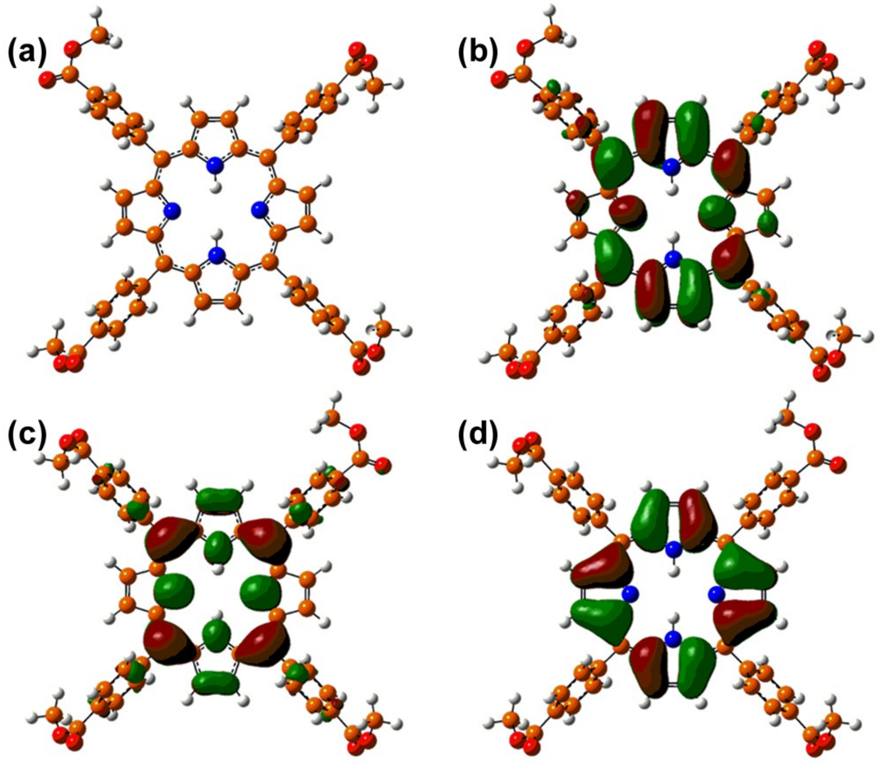

Fig. S3 (a) The model used for DFT calculation of TPPCOOMe. DFT simulated frontier orbitals of TPPCOOMe: (b) LUMO, (c) HOMO, (d) HOMO-n. The results are in consistent with the typical porphyrin frontier orbitals.[S1]

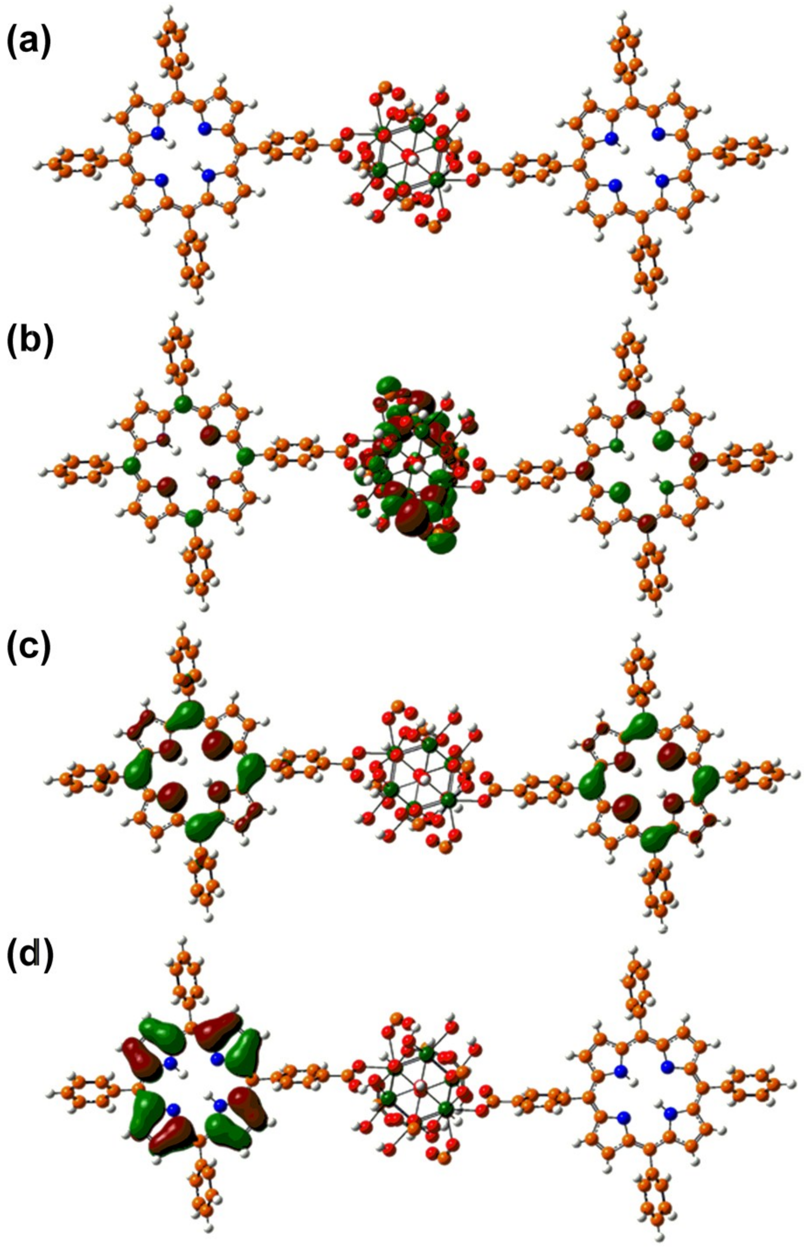

Fig. S4 (a) The PCN-222 model employed for DFT calculation. DFT simulated frontier orbitals of PCN-222: (b) LUMO, (c) HOMO, (d) HOMO-n. The similar occupied orbitals of PCN-222 to those of TPPCOOMe indicate their similar energy structure to some extent. The LUMO orbital of PCN-222 mainly locates at the Zr-oxo cluster, which indicates the possible LCCT process. Compared to TPPCOOMe, the HOMO and LUMO are separated spatially, suggesting its better electron-hole separation efficiency.

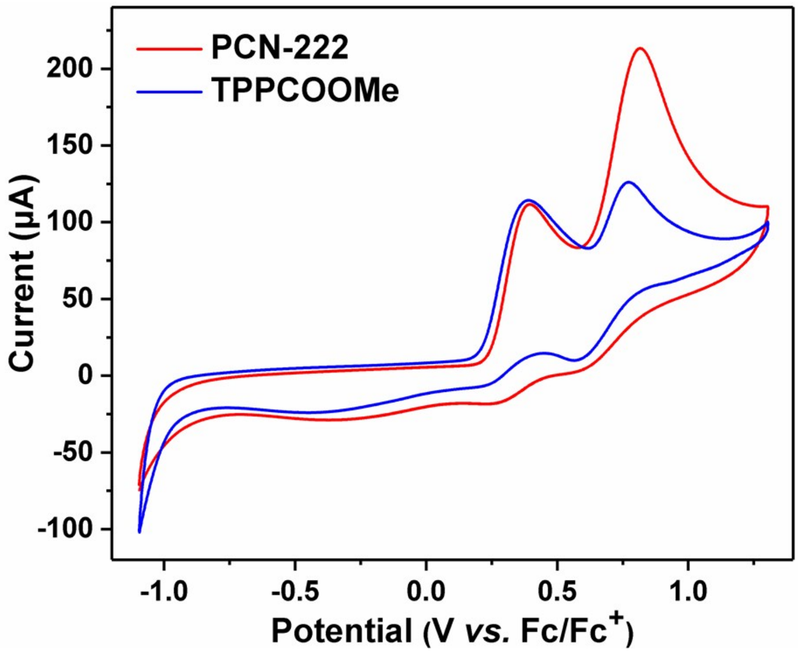

Fig. S5 Cyclic Voltammetry tests for PCN-222 and TPPCOOMe in CH_3CN solution with 0.1 M TBAF as electrolyte and ferrocene (Fc) as internal standard. Both curves show two similar oxidation peaks, indicating the existence of at least two similar occupied orbitals. The formation of the porphyrinic MOF, PCN-222, results in the obvious enhancement of the second oxidation peak, inferring that the higher electronic density at the secondary occupied orbital will be beneficial to the benzylamine oxidation.

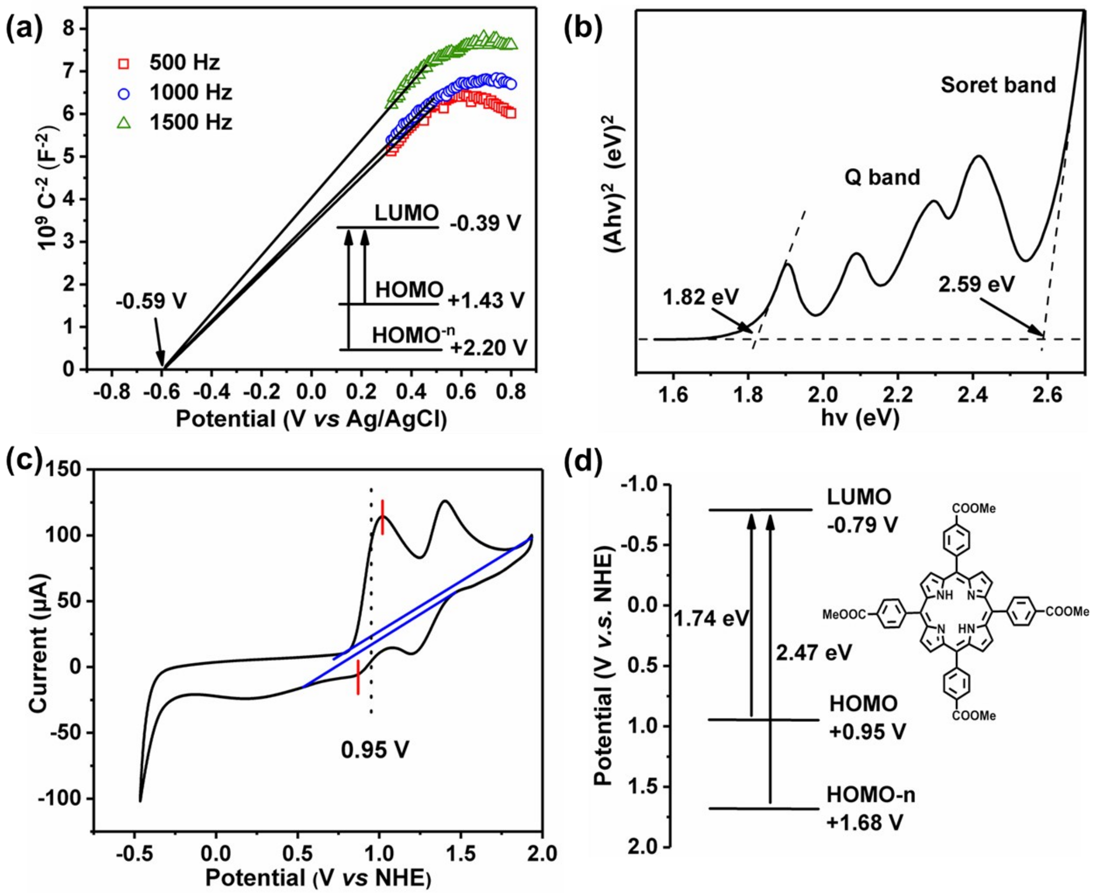

Fig. S6 (a) Mott-Schottky plots for PCN-222 (inset: the energy diagram of the HOMO-n, HOMO and LUMO levels). (b) Tauc plot for PCN-222. (c) Cyclic Voltammetry to determine the HOMO of molecular TPPCOOMe.[S2] (d) Energy diagram of TPPCOOMe. HOMO level was determined based on cyclic voltammogram results, and HOMO-LUMO gaps were estimated from the onset of the UV-Vis spectrum (see Fig. S2b).[S3]

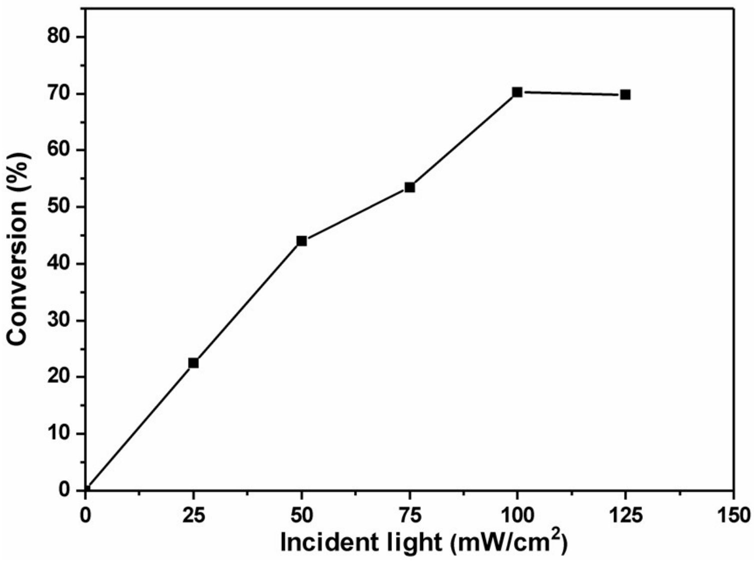

Fig. S7 The conversion *vs* light intensity in the photocatalytic oxidation of benzylamine over PCN-222. All reactions were conducted under the optimized conditions and each point was obtained at the reaction time length of 30 min.

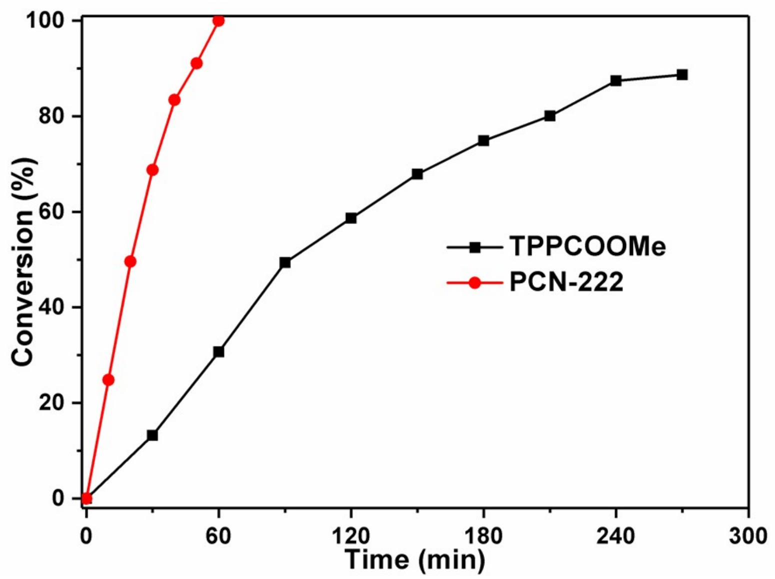

Fig. S8 Time courses of oxidative coupling of benzylamine over PCN-222 and TPPCOOMe under visible light irradiation (>420 nm). Reaction conditions: 0.1 mmol benzylamine, 5 mg PCN-222 or 3.6 mg TPPCOOMe, 100 mW/cm^2 Xe lamp, 3 mL CH_3CN, and air ballon.

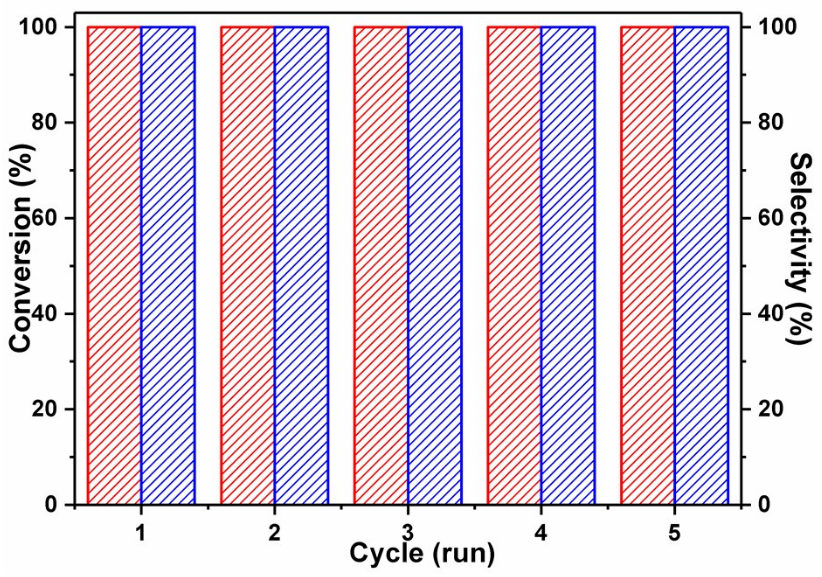

Fig. S9 Recycling performance of PCN-222 toward the photocatalytic oxidative coupling of benzylamine.

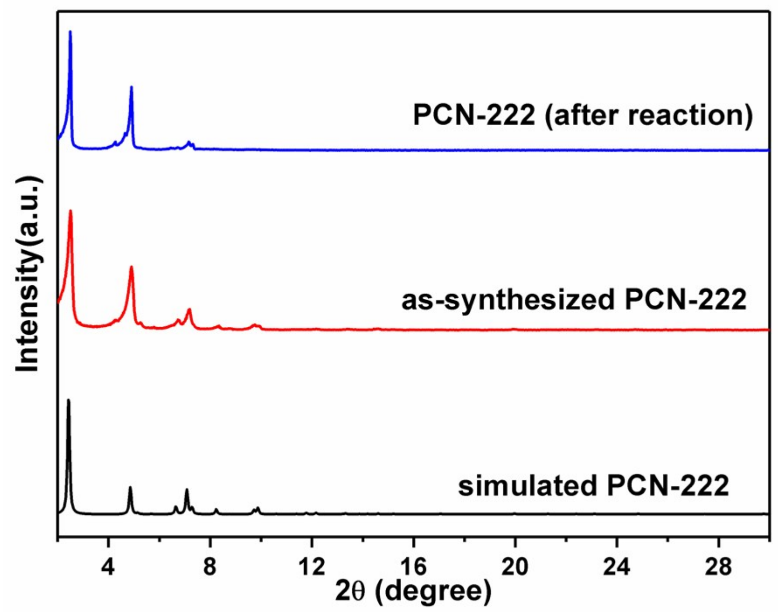

Fig. S10 PXRD patterns for simulated PCN-222 and experimental PCN-222 before and after catalytic reaction.

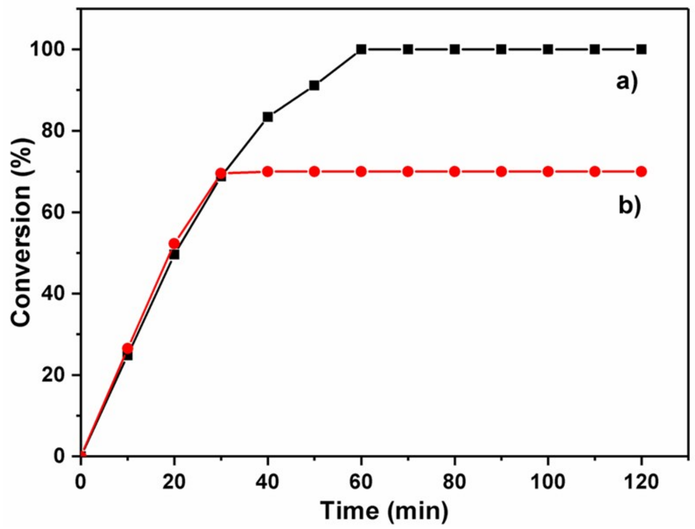

Fig. S11 Leaching test for the oxidative coupling of benzylamines over PCN-222 under optimized reaction conditions. After 30 min of the reaction, the catalyst was filtered out whereas the filtrate was further reacted under identical conditions: a) the common catalytic process and b) hot filtration test.

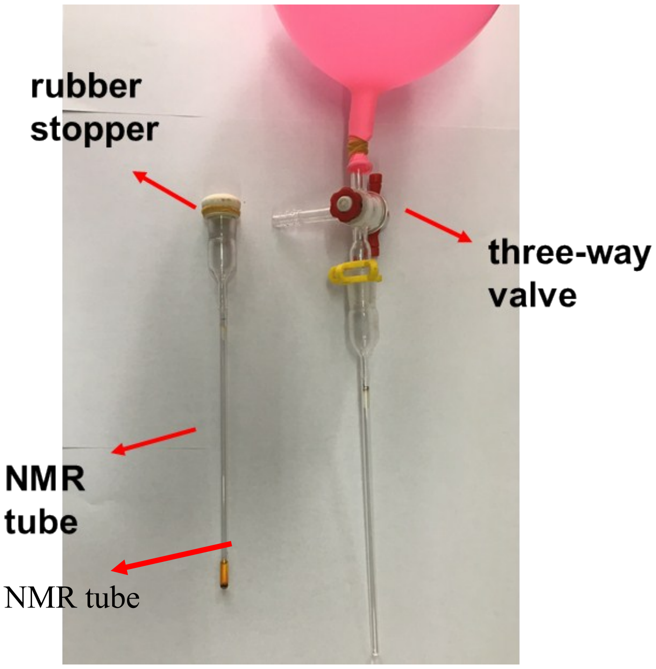

Fig. S12 The picture of the homemade testing tube for *in situ* ESR experiments. The liquid and gas additives can be injected by the syringe through the rubber stopper without disturbing the gas environment. Gas additives can be introduced by rotating the three-way valve as well. Gas or benzylamine can be introduced very quickly without moving the position and "*in situ*" results can be achieved.

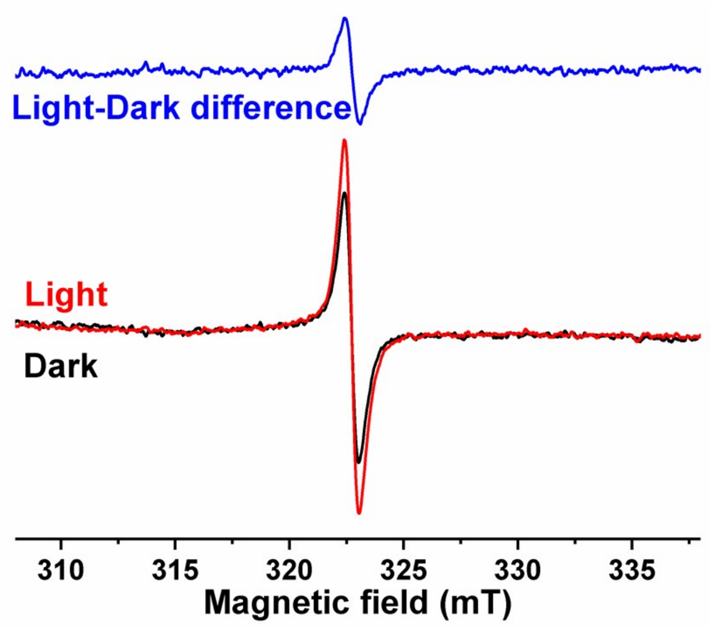

Fig. S13 ESR detection of porphyrin π-cation radical in PCN-222.

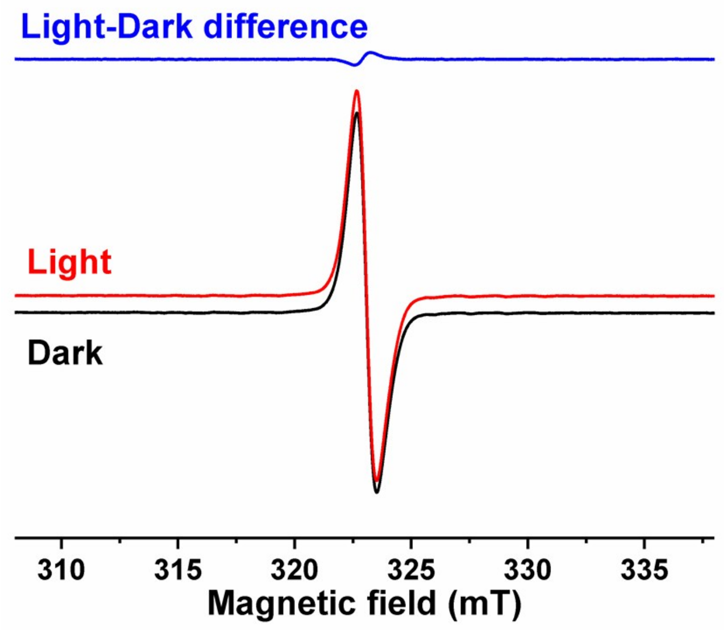

Fig. S14 ESR detection of porphyrin π-cation radical in TPPCOOMe.

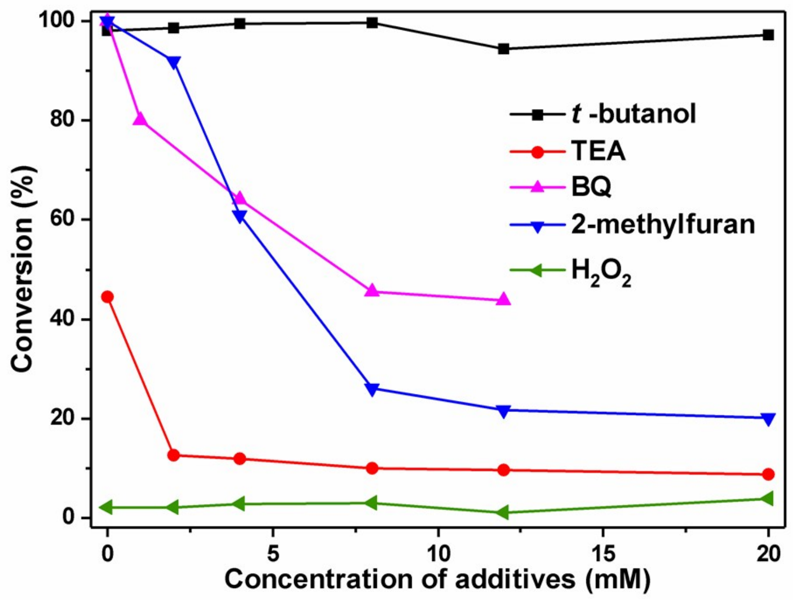

Fig. S15 Concentration influence of different additives on benzylamine oxidative coupling over PCN-222. The hole scavenger TEA was introduced under N_2 atmosphere to rule out the disturbance of active oxygen species from air.

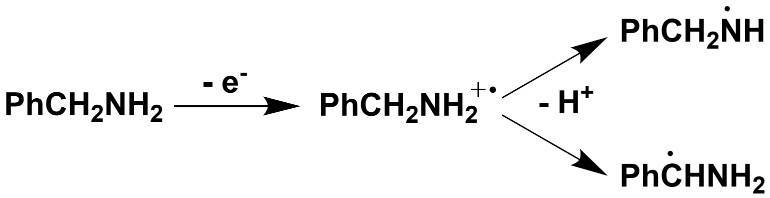

Fig. S16 Schematic showing the electron transfer from benzylamine and subsequent dehydrogenation of benzylamine radical cation to give two neutral radical species, benzyl-type carbon-centered ($PhCH^{\bullet}NH_2$) and nitrogen-centered ($PhCH_2NH^{\bullet}$) radicals. They both promote the further benzylamine oxidation reaction to afford the target product imine.[S4]

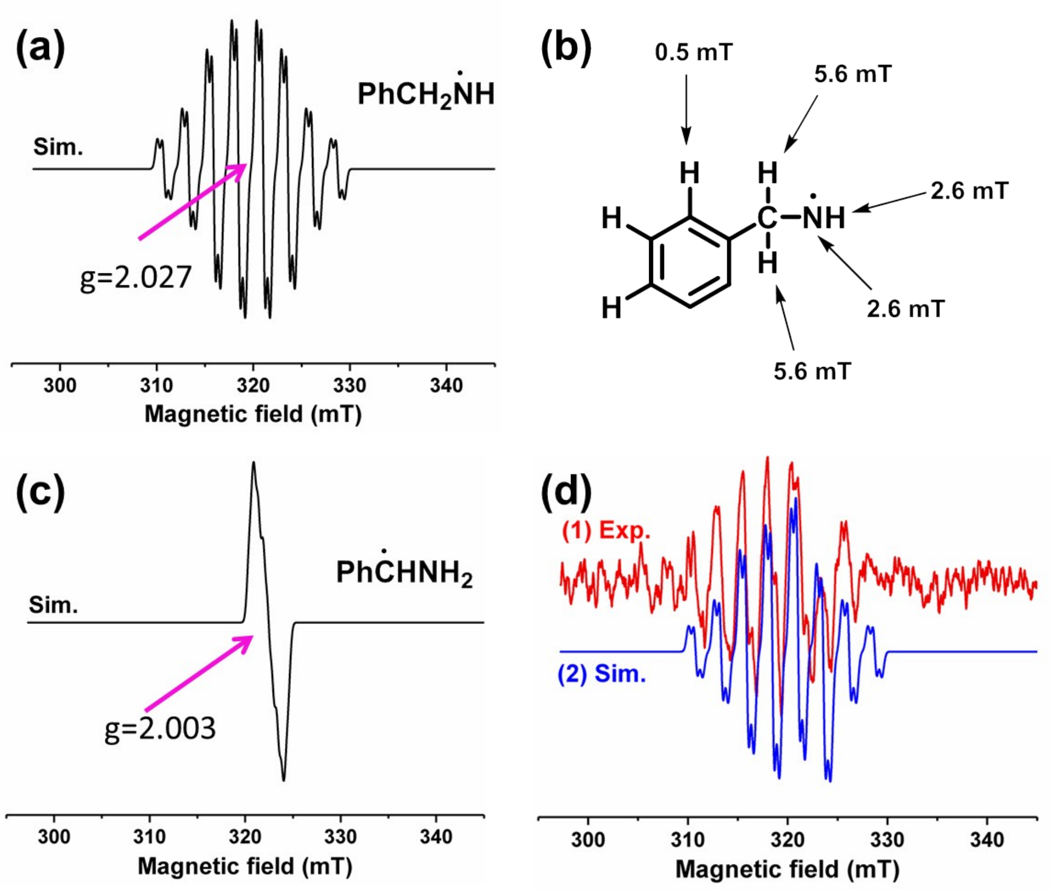

Fig. S17 (a) Simulated spectrum of nitrogen-centered radical ($PhCH_2NH^{\bullet}$) and (b) the hyperfine coupling constants. (c) Simulated spectrum of carbon-centered radical ($PhCH^{\bullet}NH_2$) according to the reference.[S5] (d) The ESR spectra of (1) experimental result after baseline correction and (2) the overlapped signals of $PhCH_2NH^{\bullet}$ and $PhCH^{\bullet}NH_2$ simulated by computer.

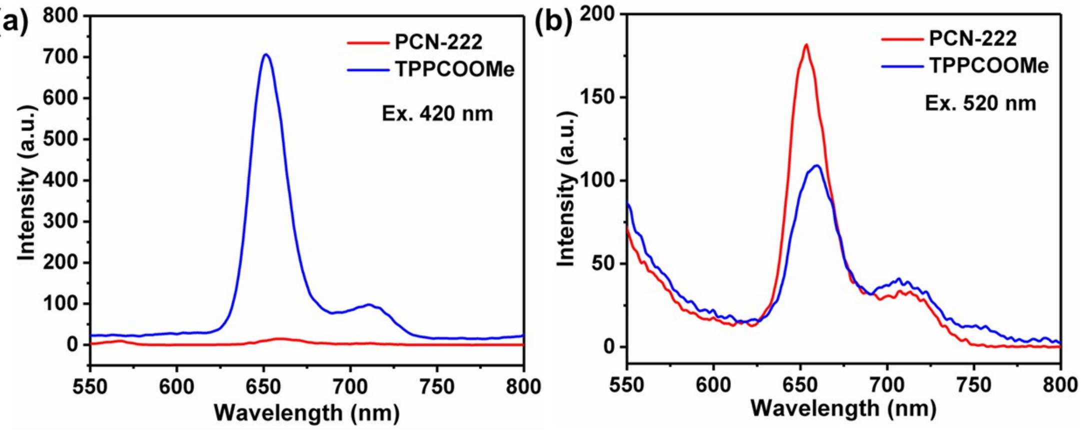

Fig. S18 Steady-state Photoluminescence (PL) spectra of PCN-222 and TPPCOOMe under excitation at (a) 420 nm and (b) 520 nm, respectively. In sharp contrast to TPPCOOMe, the PL emission of PCN-222 is almost quenched upon the excitation of Soret band (λ = 420 nm), while the intensity is higher than that of TPPCOOMe upon the excitation of Q band (λ = 520 nm).

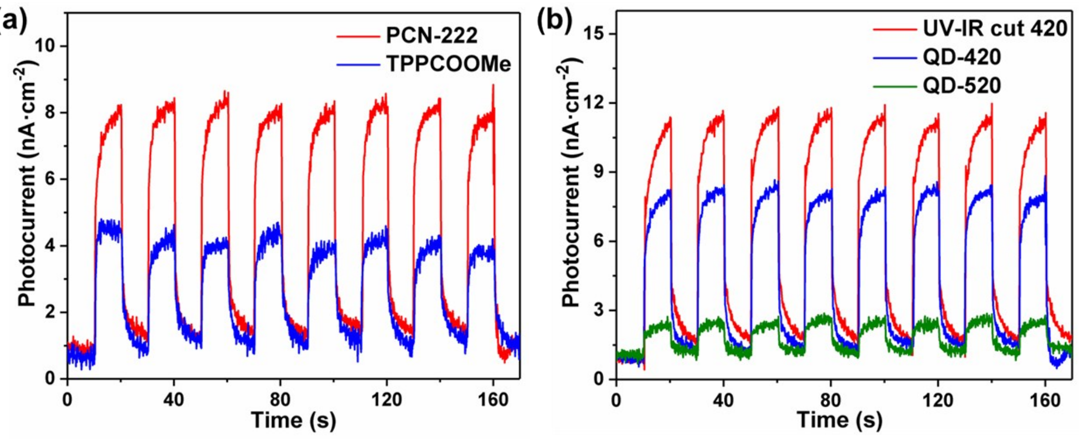

Fig. S19 (a) Photocurrent response of PCN-222 and TPPCOOMe with a fixed content of porphyrin unit under visible light (420 nm < λ < 800 nm) irradiation. (b) Photocurrent responses of PCN-222 under light irradiation by Xe lamp with a fixed output power while different wavelengths, where QD means the single wavelength irradiation with optical filter. The Q band shows much lower photocurrent response, indicating that the Q band gives much lower contribution towards charge transfer.

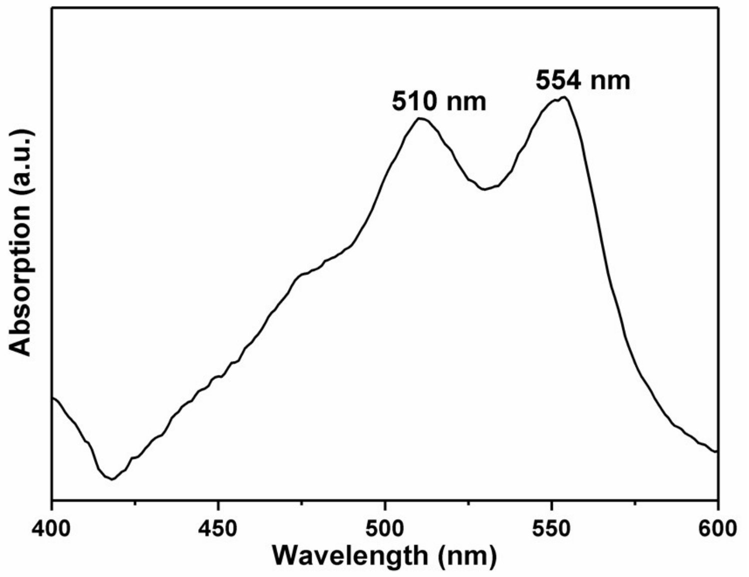

Fig. S20 Detection of H_2O_2 in the filtrate of photocatalytic aerobic oxidative coupling of benzylamine over PCN-222 (after 1 h reaction), based on a DPD/POD method.

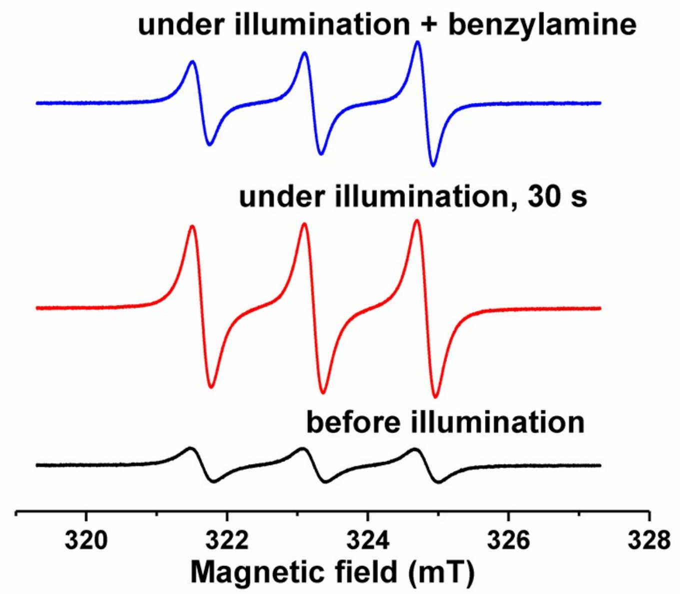

Fig. S21 ESR detection of $^1O_2$ generated by PCN-222 with 4-oxo-TMP as $^1O_2$ trapping agent. The appearance of $^1O_2$ signal before illumination might be due to the inevitable oxidation of the 4-oxo-TMP upon exposure to air.

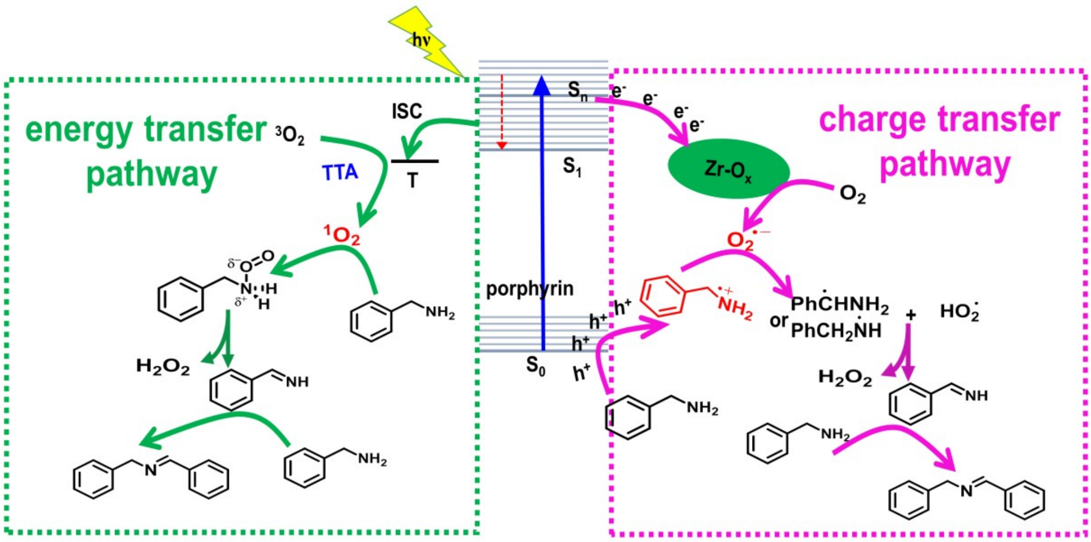

Fig. S22 The complete reaction mechanism in the aerobic oxidative coupling of benzylamine over PCN-222, via charge transfer and energy transfer,[S6] respectively.

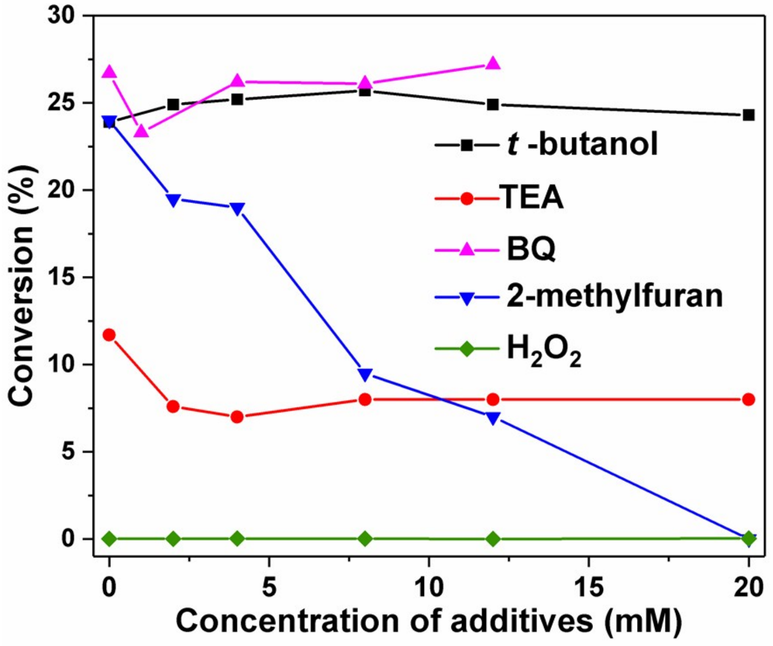

Fig. S23 Concentration influence of different additives on the oxidative coupling of benzylamine over TPPCOOMe. The hole scavenger TEA was introduced under N_2 atmosphere to rule out the disturbance of active oxygen species from air.

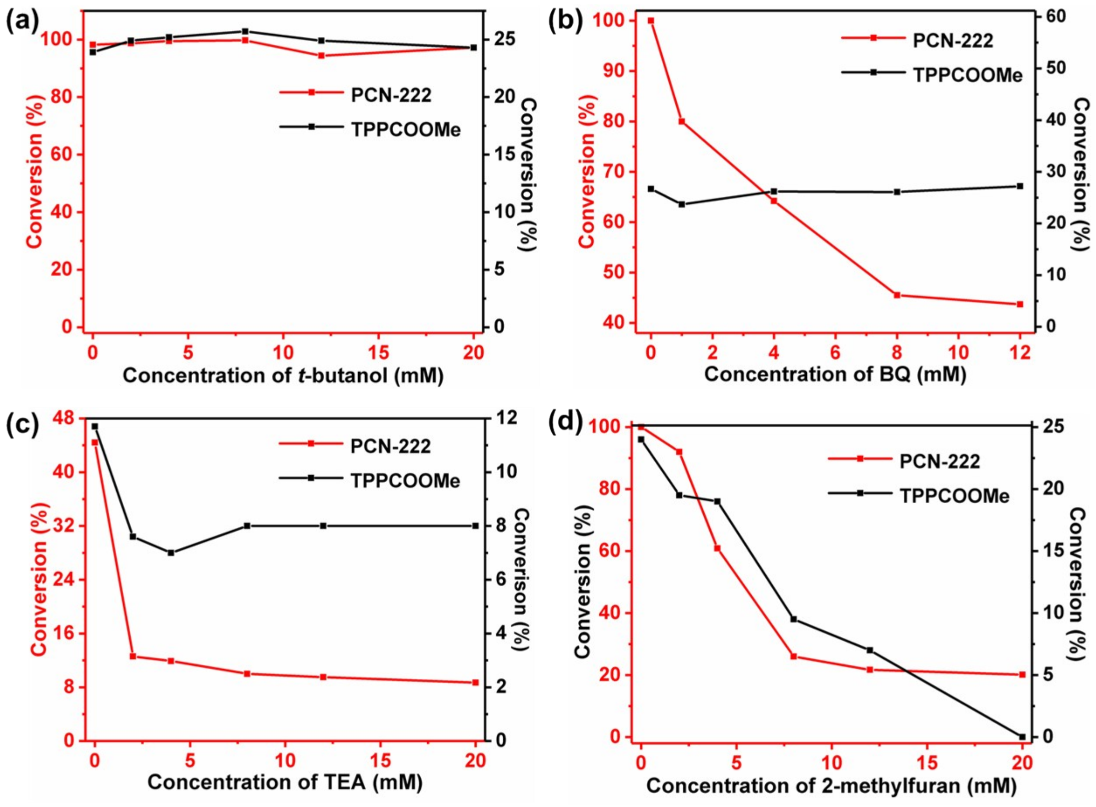

Fig. S24 The concentration influence of different additives on oxidative coupling of benzylamine over PCN-222 and TPPCOOMe, respectively. (a) •OH scavenger (*t*-butanol); (b) $O_2^{\bullet-}$ inhibitor (BQ); (c) hole scavenger (TEA); (d) $^1O_2$ scavenger (2-methylfuran). The scavenging experiments of hole scavenger were conducted under N_2 atmosphere to rule out the disturbance of active oxygen species from air.

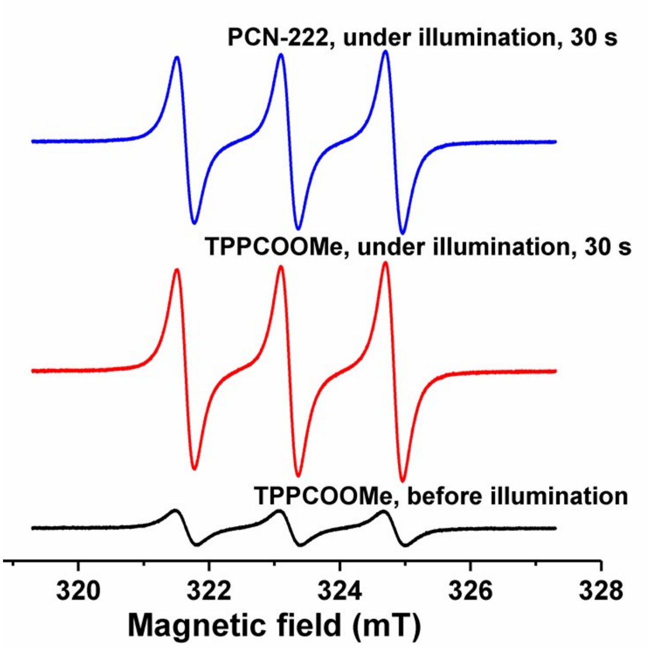

Fig. S25 ESR detection of the reactive oxygen species $^1O_2$ generated by TPPCOOMe.

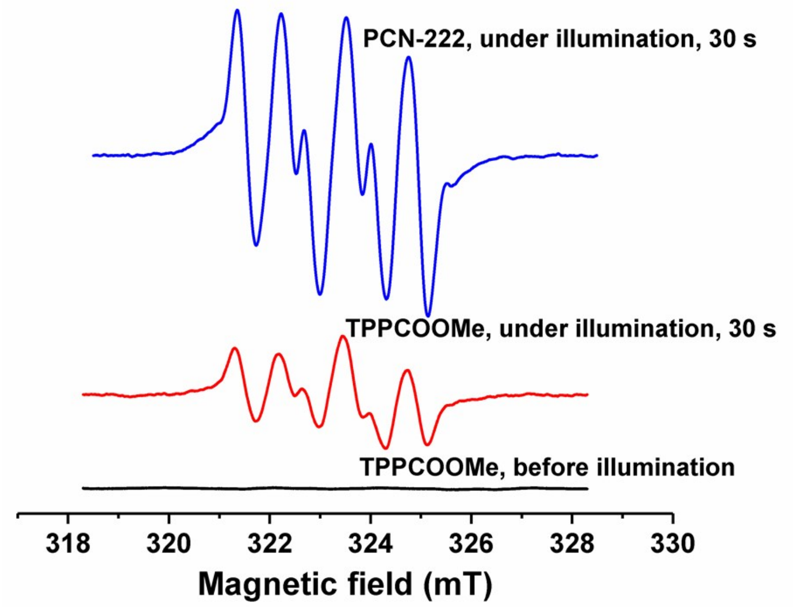

Fig. S26 ESR detection of the reactive oxygen species $O_2^{\bullet-}$ generated by TPPCOOMe and PCN-222.

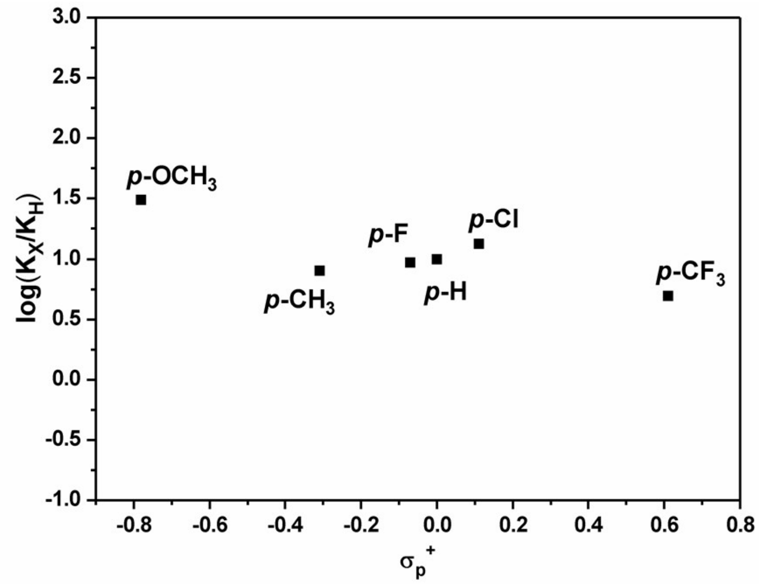

Fig. S27 Hammett plot for the photocatalytic oxidative coupling of *para*-substituted benzylamines over TPPCOOMe. The ratio of log(k_x/k_h) was obtained by the conversion with reaction time length of 30 min. Reaction conditions: 0.1 mmol of benzylamine, 3.6 mg of TPPCOOMe (equivalent amount of porphyrin motif in PCN-222), visible light (100 mW/cm^2, Xe lamp cutoff below 420 nm), 3 mL of acetonitrile and open air conditions.

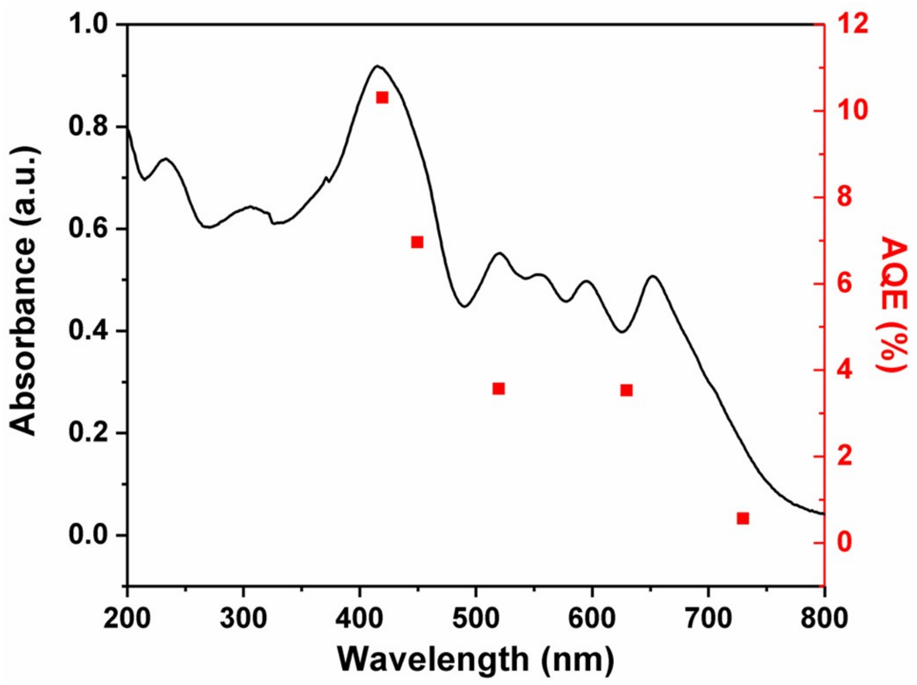

Fig. S28 Apparent quantum efficiency (AQE) of PCN-222 at different wavelengths, along with its UV–Vis absorbance spectrum. Both Soret band and Q band show obvious activity. AQE was calculated by the following equation.

$$\text{AQE} = \frac{\text{Number of products}}{\text{Number of incident photons}} \times 100\%$$

The light intensity with different optical single-wavelength filter ($\lambda_0 \pm 20$ nm) was kept as 5 mW/cm^2 by a spectroradiometer.

References

S1 M. Gouterman, *J. Mol. Spectrosc.,* 1961, **6**, 138-163.

S2 V. V. Pavlishchuk and A. W. Addison, *Inorg. Chim. Acta*, 2000, **298**, 97-102.

S3 T. Toyao, M. Saito, S. Dohshi, K. Mochizuki, M. Iwata, H. Higashimura, Y. Horiuchi and M. Matsuoka, *Chem. Commun.*, 2014, **50**, 6779-6781.

S4 R. V. Hoffman and R. Cadena, *J. Am. Chem. Soc.*, 1977, **99**, 8226-8232.

S5 K. Ohkubo, T. Nanjo and S. Fukuzumi, *Bull. Chem. Soc. Jpn.*, 2006, **79**, 1489-1500.

S6 E. Baciocchi, T. D. Giacco, O. Lanzalunga and A. Lapi, *J. Org. Chem.*, 2007, **72**, 9582-9589.
